# LC-ESI/MS/MS analysis of neonicotinoids in urine of very low birth weight infants at birth

**DOI:** 10.1371/journal.pone.0219208

**Published:** 2019-07-01

**Authors:** Go Ichikawa, Ryota Kuribayashi, Yoshinori Ikenaka, Takahiro Ichise, Shouta M. M. Nakayama, Mayumi Ishizuka, Kumiko Taira, Kazutoshi Fujioka, Toshimi Sairenchi, Gen Kobashi, Jean-Marc Bonmatin, Shigemi Yoshihara

**Affiliations:** 1 Department of Pediatrics, Dokkyo Medical University School of Medicine, Mibu, Tochigi, Japan; 2 Laboratory of Toxicology, Department of Environmental Veterinary Sciences, Faculty of Veterinary Medicine, Hokkaido University, Sapporo, Hokkaido, Japan; 3 Water Research Group, Unit for Environmental Sciences and Management, North-West University, Potchefstroom, North West, South Africa; 4 Department of Anesthesiology, Tokyo Women’s Medical University Medical Center East, Arakawa-ku, Tokyo, Japan; 5 Albany College of Pharmacy and Health Sciences, Albany, New York, United States of America; 6 Department of Public Health, Dokkyo Medical University School of Medicine, Mibu, Tochigi, Japan; 7 Centre National de la Recherche Scientifique, Centre de Biophysique moléculaire, Orléans, France; Universidade do Estado do Rio de Janeiro, BRAZIL

## Abstract

**Objectives:**

Neonicotinoid insecticides are widely used systemic pesticides with nicotinic acetylcholine receptor agonist activity that are a concern as environmental pollutants. Neonicotinoids in humans and the environment have been widely reported, but few studies have examined their presence in fetuses and newborns. The objective of this study is to determine exposure to neonicotinoids and metabolites in very low birth weight (VLBW) infants.

**Methods:**

An analytical method for seven neonicotinoids and one neonicotinoid metabolite, *N*-desmethylacetamiprid (DMAP), in human urine using LC-ESI/MS/MS was developed. This method was used for analysis of 57 urine samples collected within 48 hours after birth from VLBW infants of gestational age 23–34 weeks (male/female = 36/21, small for gestational age (SGA)/appropriate gestational age (AGA) = 6/51) who were admitted to the neonatal intensive care unit of Dokkyo Hospital from January 2009 to December 2010. Sixty-five samples collected on postnatal day 14 (M/F = 37/22, SGA/AGA = 7/52) were also analyzed.

**Results:**

DMAP, a metabolite of acetamiprid, was detected in 14 urine samples collected at birth (24.6%, median level 0.048 ppb) and in 7 samples collected on postnatal day 14 (11.9%, median level 0.09 ppb). The urinary DMAP detection rate and level were higher in SGA than in AGA infants (both p<0.05). There were no correlations between the DMAP level and infant physique indexes (length, height, and head circumference SD scores).

**Conclusion:**

These results provide the first evidence worldwide of neonicotinoid exposure in newborn babies in the early phase after birth. The findings suggest a need to examine potential neurodevelopmental toxicity of neonicotinoids and metabolites in human fetuses.

## Introduction

Neonicotinoid insecticides (neonicotinoids) are neurotoxicants with nicotinic acetylcholine receptor (nAChR) modulator action [1–3]. Neonicotinoids were first introduced on the market in the mid-1990s, and now are the most widely used class of insecticides worldwide, both for seed dressings of crops (e.g. maize, oil-seed rape, cotton, and soybeans) and for spraying on rice paddies, fruits, vegetables, tea leaves, cocoa, and coffee beans [3, 4]. In Japan, seven neonicotinoids were registered as pesticides up to 2002, with 70.3 tons of imidacloprid, 53.8 tons of acetemiprid, 8.0 tons of nitenpyram, 21.4 tons of thiacloprid, 34.4 tons of thiamethoxam, 64.2 tons of clothianidin, and 156.8 tons of dinotefuran shipped in 2009 [5,6]. Three more insecticides with nAChR modulator action have recently been registered: flupyradifurone in 2015, and sulfoxaflor and triflumezopyrim in 2018 [7]. These three molecules are also considered to be neonicotinoids because of their similarity in molecular structure and neuronal effects to those of the original seven neonicotinoids [8].

Since the mid-2000s, many studies have shown that neonicotinoids may have negative effects on non-targeted invertebrates, in particular on honeybees and bumblebees [1,4,8,9–13]. This evidence has led to prohibition of outside use of three neonicotinoids, imidacloprid, clothianidin and thiamethoxam, in the EU since 2013 [14–17] and a total ban on outside use of imidacloprid, clothianidin, thiamethoxam, thiacloprid, and acetamiprid in France in 2019 [18].

Neonicotinoids may also have negative effects on vertebrates [13, 19], including wild birds [20], bats [21], and white-tailed deer [22]. Recent *in vitro* studies have revealed multiple toxicity of neonicotinoids at a low dose, including neurotoxicity of imidacloprid at 0.77 mg/L [23], immunotoxicity of clothianidin at 0.1 mg/L [24], endocrine toxicity of imidacloprid at 0.03 mg/L and thiacloprid at 0.08 mg/L [25], and genotoxicity caused by oxidative stress [26]. A few *in vivo* studies have shown neurodevelopmental toxicity in rodents by imidacloprid 0.5 mg/kg/day, and acetamiprid 1 mg/kg/day, and neurotoxicity with clothianidin 10 mg/kg/day [27–29]. These levels are the same or lower than the no-observed-adverse-effect levels (NOAELs) of 5.7 mg/kg/day for imidacloprid, 7.1 mg/kg/day for acetamiprid, and 9.7 mg/kg/day for clothianidin [30–32]. Several reports suggest that subacute and chronic exposure to neonicotinoids such as acetamiprid and thiamethoxam may be toxic in humans [33,34], and acute high dose exposure of imidacloprid [35], acetamiprid [36, 37], and thiacloprid [38,39] can be lethal. Neonicotinoids are well absorbed by humans after oral intake and are mainly excreted in urine [40–42]. These molecules cross the human blood brain barrier [43], and some neonicotinoids have toxic metabolites, such as desnitroimidacloprid, which has a mammalian nAChR agonistic activity that is as high as that of nicotine [44].

Current reports of neonicotinoid food contamination at less than the maximum residual dose are increasing. Japanese non-organic green tea leaves are contaminated by dinotefuran with imidacloprid, acetamiprid, clothianidin, thiacloprid and thiamethoxam [45]. In the EU, acetamiprid was detected in 10% of apples, imidacloprid in 15.8% of lettuces, and thiacloprid in 11.4% of strawberries [46]. In the US, acetamiprid was detected in 13.4% of fruits and imidacloprid in 19.9% of vegetables in 1999–2015 [47]. A particularly toxic neonicotinoid metabolite, desnitro-imidacloprid, has been detected in drinking water in the US [48]. Unlike most other pesticides, neonicotinoids cannot be washed off food prior to consumption due to the characteristics of the plant [47].

Frequent detection of neonicotinoids and their metabolites in urine and hair have been reported for the general population [42,49–52], but this has not been investigated in fetuses and newborn babies, despite their potentially high sensitivity to these chemicals [53]. Developing cerebral vessels in infants are more fragile than those in adults and more vulnerable to drugs, toxins, and pathological conditions, which may cause cerebral damage and subsequent neurological disorders [54]. Many adult functions, including effective tight junctions, are not developed in the embryonic brain and some transporters are more active during development than in adults [55,56].

In Japan in 2009, the incidence of low-birth-weight (LBW) infants (<2500 g at birth) was 9.6%, and that of very (V)LBW infants (<1500 g) was 0.74% [57]. LBW infants are classified as small for gestational age (SGA), indicating those who are smaller in size, with weight below the 10^th^ percentile for gestational age; or appropriate for gestational age (AGA), for those who are appropriate in size, with weight and head circumference in the range from the 10^th^ to 90^th^ percentile. In general, neurological development in SGA infants is worse than that in AGA infants [58]. In addition to body weight, head circumference is used as an index of development, and the head circumference SD score can be calculated by the lambda-mu-sigma method using LMS chart-maker. This score is the international standard for newborn size for each gestational age based on data from the Newborn Cross-Sectional Study, which conforms at the population and individual levels to the prescriptive approach used in the WHO Multicentre Growth Reference Study [59]. A low head circumference SD score is related to neurodevelopment delay [58].

In this study, we developed an analytical method for seven neonicotinoids and one neonicotinoid metabolite in human urine. Then we explored exposure to neonicotinoids and metabolites in VLBW infants born in 2009–2010 in the early stage after birth to examine whether neonicotinoids can be transferred to fetuses. These infants are not usually fed with milk for 48 hours at birth. The relationships of detection of neonicotinoids with body weight and head circumference SD scores were also examined.

## Subjects and methods

### Subjects and sample collection

The subjects were infants born at a gestational age of 23 to 32 weeks and a birth weight of 500–1,500 g who were admitted to the NICU of Dokkyo Medical University Hospital from January 2009 to December 2010. Infants with chromosomal abnormalities, external deformities and life-threatening diseases were excluded. After obtaining approval from the ethics committee of Dokkyo Medical University (approval no. 25042) and informed consent from the infants’ parents, urine samples were collected on postnatal days (PNDs) 1 to 2 (within 48 h after birth) and PND 14 using cotton balls or a urine sampling bag, and the samples were stored at -80°C. The cotton ball was applied to the absorbent core in the diaper and collected after it was immersed in urine. The current study was performed using urine samples collected from these infants for a previous study. We obtained new approval from the ethics committee of Dokkyo Medical University (approval no.29008) and gave an explanation to the infants’ parents by posting a notice and through an opt-out method, which was also approved by the ethics committee of Dokkyo Medical University.

### Chemicals

Acetamiprid, dinotefuran, imidacloprid, nitenpyram and thiacloprid were purchased from Kanto Chemical Corp. (Tokyo, Japan). Clothianidin, clothianidin-d3, dinotefuran-d3, imidacloprid-d4, thiacloprid-d4, thiamethoxam-d4, and *N*-desmethylacetamiprid (DMAP) were purchased from Sigma-Aldrich (St. Louis, MO, USA). Hydroxyimidacloprid was purchased from Hypha Discovery (Slough, UK). Acetamiprid-d6 and nitenpyram-d3 were purchased from Hayashi Pure Chemical Ind. (Osaka, Japan). Thiamethoxam was purchased from Dr. Ehrenstorfer. Acetonitrile, dichloromethane formic acid, ammonium acetate and distilled water were all HPLC grade and were purchased from Kanto Chemical.

#### Urine sample preparation and analysis

Urine was thawed, stirred, and allowed to stand for some time and the supernatant was used. Purification of urine was performed by solid phase extraction (SPE). A volume of 100 μL of internal standard mixture (each 10 ppb) was added to 100 μL of each urine sample, and then 2800 μL of distilled water was added to the sample. Two types of SPE cartridges were used for purification: an InertSep Pharma SPE column (60 mg/3 ml) (GL Science, Tokyo, Japan) pre-conditioned with 3 mL of an acetonitrile/dichloromethane (1/1) mixture followed by 3 ml of distilled water; and an InertSep PSA SPE column (100 mg/1ml) (GL Science) pre-conditioned with 1 mL of the acetonitrile/dichloromethane (1/1) mixture. Prepared samples were loaded on the pre-conditioned InertSep Pharma and washed with 0.5 mL of distilled water. The InertSep Pharma (top) was combined with the InertSep PSA (bottom) and 3 ml of the acetonitrile/dichloromethane (1/ 1) mixture were used to elute the target chemicals. After concentrating and dry-solidifying with a centrifugal concentrator (CVE-200D with UT-2000, Eyela, Tokyo, Japan), the samples were reconstituted with 100 μL of 3% methanol in distilled water and transferred to vials for analysis. Seven neonicotinoids and DMAP were analyzed in each sample. Recovery rates and LOQs are shown in Table 1.

**Table 1:** Properties of target neonicotinoids and metabolites.

Neonicotinoids	MRM^a^ (*m*/*z*)	RT^b^ (min)	Recovery rate (%)	LOQ^c^ (ng/ml)
Dinotefuran	203.00>129.10	8.2	92.6 ±2.8	0.125
Nitenpyram	271.00>126.05	8.9	88.6 ±4.6	0.5
Thiamethoxam	291.90>211.00	14.0	116.7 ±7.9	0.125
*N*-Desmethylacetamiprid (DMAP)	208.90>126.05	15.2	87.6 ±5.4	0.05
Clothianidin	249.90>132.05	16.1	91.8 ±3.7	0.125
Acetamiprid	223.00>126.00	16.2	80.2 ±2.9	0.05
Imidacloprid	256.00>209.05	17.3	87.0 ±2.7	0.5
Thiacloprid	252.90>126.05	19.1	92.9 ±1.8	0.05

^a^ MRM: multiple reaction monitoring;

^b^RT: Retention time;

^c^LOQ: limit of quantification

A LC-ESI/MS/MS system (Agilent 6495B, Agilent Technologies, Santa Clara, CA, USA) equipped with a Kinetex Biphenyl column (2.1 mm ID × 100 mm, ϕ2.6 μm, Phenomenex, Torrance, CA, USA) was used for sample analysis. Solvents A and B used for HPLC analysis were 0.1% formic acid + 10 mM ammonium acetate in aqueous solution and 0.1% formic acid + 10 mM ammonium acetate in methanol, respectively. The gradient was programmed as: *t* = 0 to 1 min: 5% B, *t* = 6 min: 95% B, *t* = 6 to 8 min: 95% B. The column oven temperature and flow rate were 60°C and 0.5 ml/min, respectively. For mass spectrometry, multiple reaction monitoring (MRM) was programmed (Table 1). The recovery rate of each neonicotinoid and its metabolites ranged from 80% (acetamiprid) to 117% (thiamethoxam). The reproducibility of the analysis system was confirmed in the same or plural analyses, with a relative standard deviation (RSD) of 10% for all the compounds.

#### Quantitation of neonicotinoids and their metabolites

Seven neonicotinoids and DMAP (Table 1) were analyzed in each sample. Six compounds were used as internal standards. The precursor and product ions are shown in Table 1. Quantification of the neonicotinoids and metabolites was carried out by the internal standard method. Five calibration points were set at 0.5, 1.25, 2.5, 3.75 and 5 ppb, whereas the internal standard was used to 5 ppb at all calibration points.

#### Quality control and quality assurance

A mixture of six deuterium-labeled neonicotinoids was spiked into samples as an internal standard prior to sample preparation and extraction. Quantitation was performed using five calibration points and the average coefficients of determination (*r*^*2*^) for the calibration curves were ≥0.995. The analytical method was checked for precision and accuracy. Limits of detection (LODs) were calculated based on 3*SD*/*S* (*SD* is the standard deviation of the response of seven replicate standard solution measurements and *S* is the slope of the calibration curve). Recovery rates and LOQs (ng/mL) of the analytes are given in Table 1.

### Statistical analysis

IBM SPSS Statistics 23 was used for statistical analysis. A Fisher exact test was used to compare the DMAP detection rates. A Wilcoxon rank sum test for non-parametric data was used to compare DMAP concentrations. Spearman rank correlation coefficient analysis was used to compare the DMAP level and infant physique index (length SD score, height SD score, and head circumstance SD score). The significance level was set at P = 0.05.

## Results

Fourteen of the 130 urine samples collected from 65 subjects could not be analyzed due to insufficient volume, and thus, the final analysis included 116 samples: 57 collected on PND 1–2 (within 48 h after birth) and 59 on PND 14. The background of the subjects, including physical status, is shown in Table 2.

**Table 2:** Characteristics of infants on postnatal days (PNDS) 1–2 and 14.

Item	PND 1–2	PND 14	P
Number of samples	57	59	
Gestational age (weeks)	27 (28, 23–34)	27 (28, 23–34)	0.84
Sex, male	36 (63%)	37 (63%)	0.92
Birth weight (g)	1012 (982,515−1474)	967 (926,515–1474)	0.69
Birth weight SD score	-0.6 (-0.6,-3.1−2.0)	-0.6 (0.1,-3.3−2.0)	0.75
Birth length (cm)	35.1 (35, 28−41)	34.7 (35, 28−41)	0.99
Birth length SD score	-0.4 (-0.4,-2.6−1.7)	-0.4 (-0.4,-2.6−1.7)	0.77
Head circumference (cm)	25.3 (25.5,19.8−29.5)	25.0 (24.5,19.8−29.5)	0.89
Head circumference SD score	0.1 (0.2,-1.8−1.4)	0.1(0.3,-1.8−1.4)	0.88
Small for gestational age	6 (11%)	8 (14%)	0.32
Apgar score 5 min	7.6 (8, 1−10)	7.5(7, 1−10)	0.75
Cesarean section	35 (61%)	38 (64%)	0.51

Data are shown as a mean (median, range) or as n (%)

Dinotefuran was detected in one sample and one of the 20 neonicotinoid metabolites, DMAP, was also detected. The concentration of dinotefuran was 0.4 ppb (PND 1–2). DMAP was detected in 14/57 PND 1–2 samples (24.6%) (median level 0.048 ppb, range: 0.01–0.68 ppb), and in 7/59 PND 14 samples (11.9%) (median level 0.09 ppb, range: 0.01–0.47 ppb), with no significant difference in the detection rate (p = 0.09) or level (p = 0.09) between PND 1–2 and PND 14 samples (Table 3).

**Table 3:** Detection of DMAP in urine of VLBW infants on postnatal days (PNDS) 1–2 and 14.

Item	PND 1–2	PND 14	p
Detection rate (%) (number of samples)	24.6 (14/57)	11.9 (7/59)	0.09
Mean concentration (ppb) (median, range)	0.11 (0.048, 0.01–0.68)	0.13 (0.09, 0.01–0.47)	0.09

The DMAP detection rate was significantly higher in SGA infants than in AGA infants (42.9% vs. 14.7%, p<0.05). The mean DMAP level was similarly significantly higher in SGA infants (0.04 vs. 0.02 ppb, p<0.05) (Table 4). There was no significant difference in DMAP detection rates on PND 1–2 for infants with positive or negative head circumference SD scores (p = 0.07) (Table 5). In infants in whom DMAP was detected on PND 1–2, there were weak negative correlations between DMAP levels and birth weight SD scores (ρ = −0.37, p = 0.19), birth length SD scores (ρ = −0.36, p = 0.20), and birth head circumference SD scores (ρ = −0.23, p = 0.43), but none of these relationships were significant.

**Table 4:** Differences in detection rates and levels of DMAP in SGA and AGA infants.

Item	SGA^a^	AGA^b^	p
Detection rate (%) (number of samples)	42.9 (6/14)	14.7 (15/102)	0.005
Mean concentration (ppb) (median, range)	0.04 (0, 0–0.3)	0.02 (0, 0–0.68)	0.004

^a^SGA: small for gestational age;

^b^AGA: appropriate for gestational age

**Table 5:** Relationships of detection of DMAP on PND 1–2 and head circumference SD score.

Item	Head circumference SD score		p
	Positive	Negative	
Detection rate (%) (number of samples)	16.7 (6/36)	38.1 (8/21)	0.07 *
Mean concentration (ppb) (median, range)	0.025 (0, 0–0.68)	0.032 (0, 0–0.30)	0.07†

*p by χ^2^ test.

^†^p by Wilcoxon rank sum test.

## Discussion

This is the first report worldwide to suggest that DMAP, a toxic metabolite of acetamiprid, may transfer to fetuses at a high rate. The oral median lethal dose (LD_50_) of DMAP is 1,843 mg/kg in female rats [31]. DMAP is the most frequently detected neonicotinoid metabolite in the general Japanese population by ppb level [42], and Marfo et al. reported that DMAP is often detected in patients with symptoms of recent memory loss and finger tremor [34].

DMAP might be transferred via the placenta and accumulate in the fetus, because it was frequently detected in urine collected on PND 1–2 and the level did not increase significantly on PND 14. Acetamiprid is rapidly metabolized to DMAP after oral administration in healthy male adults [42]. Its urinary excretion half-life is 1.65 days [38]. Continuous maternal intake of acetamiprid may cause high DMAP levels in maternal blood and DMAP contamination in the fetus. There are two possible reasons why DMAP was detected at a higher rate in SGA infants. First, body composition differs between SGA and AGA infants: % body fat is lower in SGA infants and the brain volume is relatively large. Assuming that DMAP accumulates via nAChRs in the brain, more neonicotinoids may accumulate in SGA infants. A second reason is that DMAP might inhibit growth by affecting neurological development of the fetus.

The parent compound of DMAP, acetamiprid, is a common neonicotinoid in Japan that is used for a wide range of plant protection, including fruits, vegetables, tea leaves, rice paddies, turf, ornamental flowers, and pine trees. The oral LD_50_ of acetamiprid is 146 mg/kg in female rats [31]. Fatal cases of human acute intoxication have also been reported [36]. In acute intoxication by acetamiprid, nicotinic symptoms including neuronal symptoms are observed [37]. Acetamiprid has some lipophilicity (Log P_ow_ is 0.8) and is not ionized at physiological pH [31], which suggests that it may be retained in the human body, even if its receptor action is weak. There is also some evidence to suggest that acetamiprid is toxic for neurological development [27, 59–61]. It has yet to be clarified whether neonicotinoids have neurological toxicity in infants, but the safety of acetamiprid should be reviewed based on the possibility that neonicotinoids may transfer to and accumulate in fetuses at a high rate.

The limitations of this study include the small number of subjects, investigation in one region in Japan, the inclusion of VLBW infants born prematurely rather than term newborns, and the lack of examination of pesticides other than neonicotinoids. However, it is likely that exposure to neonicotinoids observed in infants born prematurely will be similar in term newborns because they experience a similar period of exposure. Further studies are needed in a larger number of subjects in various regions, but similar results are likely because the use and environmental detection rates of neonicotinoids have increased worldwide.

## Conclusion

This report provides the first evidence worldwide showing that *N-*desmethylacetamiprid (DMAP), a metabolite of acetamiprid, can be transferred to fetuses. DMAP levels were also significantly higher in SGA infants than in AGA infants. The fetal and neonatal periods are extremely important for neurological development, and further studies are needed with regard to the safety of acetamiprid due to transfer and accumulation of its metabolite in the womb.
